# Nitrogen‐Doped Ultrananocrystalline Diamond – Optoelectronic Biointerface for Wireless Neuronal Stimulation

**DOI:** 10.1002/adhm.202403901

**Published:** 2025-02-11

**Authors:** Yue Yao, Arman Ahnood, Andre Chambers, Wei Tong, Steven Prawer

**Affiliations:** ^1^ School of Physics The University of Melbourne Parkville Victoria 3010 Australia; ^2^ School of Engineering The RMIT University Melbourne Victoria 3000 Australia; ^3^ Department of Mechanical Engineering The University of Melbourne Parkville Victoria 3010 Australia

**Keywords:** nanostructured crystals, neural interfaces, neural stimulation, optoelectronics, photovoltaic, ultrananocrystalline diamonds

## Abstract

This study presents a semiconducting optoelectronic system for light‐controlled non‐genetic neuronal stimulation using visible light. The system architecture is entirely wireless, comprising a thin film of nitrogen‐doped ultrananocrystalline diamond directly grown on a semiconducting silicon substrate. When immersed in a physiological medium and subjected to pulsed illumination in the visible (595 nm) or near‐infrared wavelength (808 nm) range, charge accumulation at the device‐medium interface induces a transient ionic displacement current capable of electrically stimulating neurons with high temporal resolution. With a measured photoresponsivity of 7.5 mA W^−1^, the efficacy of this biointerface is demonstrated through optoelectronic stimulation of degenerate rat retinas using 595 nm irradiation, pulse durations of 50–500 ms, and irradiance levels of 1.1–4.3 mW mm^−2^, all below the safe ocular threshold. This work presents the pioneering utilization of a diamond‐based optoelectronic platform, capable of generating sufficiently large photocurrents for neuronal stimulation in the retina.

## Graphical abstract

Researchers demonstrate a diamond‐based, wireless biointerface capable of generating safe capacitive current under pulsed light illumination in the visible and near‐infrared light region. This biointerface is long‐lasting and the first among diamond‐based electrodes capable of optically‐controlled, non‐genetic modulation of intracellular calcium dynamics in degenerate rat retinas.

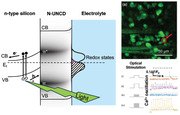

## 1 Introduction

Neurological disorders in the central nervous system are attributed in part to dysfunctional connectivity in neural networks. To study such networks, it is desirable to stimulate and monitor activities in real time using neuroprosthetics. The most mature neuroprosthetic devices approved for human use, such as deep brain stimulators^[^ ^1^ ^]^ and cochlear implants,^[^ ^2^ ^]^ rely on electrically‐driven electrodes for neuromodulation.^[^ ^3^ ^]^ Conventional electrodes for neural stimulation include materials such as platinum, iridium oxide, titanium nitride, and, more recently, poly(3,4‐ethylenedioxythiophene):poly(styrene sulfonate) (PEDOT:PSS). However, some of these electrodes exhibit significant changes in electrochemical stability under physiological conditions and during chronic implantation.^[^ ^4^ ^]^ In addition, surgical challenges arise from cumbersome device architecture, wired electrical components, and integrated processing electronics.^[^ ^5^ ^]^ In contrast, optoelectronic devices offer a promising alternative for achieving optical control of neural activity, providing high spatiotemporal resolution and a wireless stimulation modality.^[^ ^6^ ^]^ This strategy holds great potential for minimally invasive interrogation of neural networks in real time.

Recent literature reports various optically‐driven neural biointerfaces constructed from functional materials such as silicon,^[^ ^7^ ^]^ graphene,^[^ ^8^ ^]^ organic polymers,^[^ ^9^ ^]^ carbon‐nanotubes,^[^ ^10^ ^]^ and quantum dots.^[^ ^11^ ^]^ These devices are available in diverse geometrics – filaments,^[^ ^12^ ^]^ sheets,^[^ ^13^ ^]^ and open‐mesh structures^[^ ^14^ ^]^ – enhancing integration with neural tissue. For instance, Ferlauto et al. reported a photovoltaic epiretinal prosthesis utilizing organic semiconductors for wireless retinal ganglion cell (RGC) stimulation.^[^ ^15^ ^]^ This device featured a stacked configuration, including a PEDOT:PSS injection layer and a semiconductor layer made of poly(3‐hexylthiophene‐2,5‐diyl) (P3HT) and[6,6]‐phenyl‐C61‐butyric acid methyl ester (PCMB). With spatial resolution provided by 2215 channels and pixel diameters of 80–130 µm, the device enabled high spatial resolution retinal stimulation. Similarly, Parameswaran et al. developed coaxial p‐type/intrinsic/n‐type silicon nanowires (200–250 nm diameters), achieving subcellular stimulation resolution.^[^ ^16^ ^]^ Photocurrents of up to 120 pA were observed under standard irradiation conditions (532 nm laser, 8.5 mW, 10 ms pulses), and action potentials were recorded from rat dorsal root ganglion neurons (DRGs) using 1 ms light pulses at 13.5 mW laser power. More recently, Karatum et al. developed a flexible quantum dot (QD) photovoltaic biointerface capable of inducing action potentials in primary hippocampal neurons through capacitive ionic currents generated by near‐infrared light exposure.^[^ ^17^ ^]^ In these studies, either capacitive^[^ ^11^, ^15^ ^]^ or Faradaic mechanisms^[^ ^16^ ^]^ underpinned the observed electrophysiological effects. The choice of mechanisms depends on the specific applications. Few optoelectronic devices currently meet the charge density requirements for effective neurostimulation. Those that do often face limitations, including potential cytotoxicity from heavy metals in quantum dots^[^ ^11^ ^]^ and degradation under chronic conditions due to oxidation or hydration.^[^ ^16^ ^]^ These issues motivate the development of optoelectronic devices based on capacitive coupling to overcome these challenges.

Semiconducting diamonds, particularly doped diamonds, remain relatively unexplored for optoelectronic cell stimulation. From a biological perspective, diamond‐based optoelectronic devices offer two key advantages: high biocompatibility, which minimizes immunological responses and preserves signal quality at the electrode‐cell interface, and excellent stability, as diamond's impermeability to water reduces electrode degradation during chronic implantation.^[^ ^18^ ^]^ Previous work by our group demonstrated that nitrogen‐doped ultrananocrystalline diamond (NUNCD) generated a capacitive photocurrent under irradiation, making it a promising material for photochemical stimulation of excitable cells.^[^ ^19^ ^]^ The major drawback, however, was the relatively weak photoresponse compared to devices reviewed earlier. At a photoresponse of 3.75 µA W^−1^ using near‐infrared irradiation (808 nm, 41 mW mm^−2^), our earlier study reported no direct evidence that this photoresponse was sufficient to induce neuronal stimulation. Moreover, the use of high‐intensity near‐infrared light was suboptimal due to potential thermal damage or inadvertent neuronal activation via temperature‐sensitive ion channels.^[^ ^20^ ^]^ However, extending the operation of the device to visible light stimulation of neurons remains an unresolved challenge. In a subsequent study, oxygen‐terminated NUNCD substrates were shown to enhance the growth of cultured cortical neurons, promoting neuronal survival and neurite outgrowth under pulsed light stimuli with an infrared source.^[^ ^21^ ^]^ NUNCD demonstrated biocompatibility and the ability to influence neuronal gene expression and neurite outgrowth through light‐ and electrically mediated effects. However, to date, diamond‐based optoelectronic systems have been inadequate for eliciting direct retinal stimulation using physiologically safe light intensities and wavelengths. This is primarily attributed to the photocurrent being insufficient to deliver the charge density required to evoke neuronal activity.

In the present study, we describe optimization of thin‐film NUNCD optoelectronic biointerfaces that provide photocurrents sufficient for extracellular neurostimulation in vitro. In particular, we optimize the film thickness, and suggest that observed enhanced photoresponse, compared to its thicker counterparts, could be due to increased charge collection efficiency. We investigated two architectures: i) NUNCD on silicon substrate (NUNCD‐Si), and ii) NUNCD on single‐crystal diamond substrates (NUNCD‐SCD). These photovoltaic interfaces function as extracellular capacitive electrodes to facilitate neurostimulation by transducing incident light into photocarriers which are subsequently transported to the electrode‐electrolyte interface where they appear as photocurrents. Device architectures based on such materials are of particular interest because they offer the possibility of wireless operation by replacing cumbersome wiring and electronics with simple optical stimulation, thereby simplifying the processes of surgery and implantation. To carry out a systematic and quantitative study of the light‐induced electrochemical pathways, the biointerfaces were characterized using UV–vis spectroscopy to determine the optical properties of the electrode material. Following this, cyclic voltammetry and other electrochemical measurements characterized the charge storage capacity and photoresponse. Finally, we conducted calcium imaging to show light‐controlled non‐genetic modulation of intracellular calcium dynamics in degenerate rat retinas under visible light (595 nm), demonstrating the efficacy of the biointerface to photoelectrochemically excite neurons within the ocular safety limit. Overall, our biointerface generated depolarizing and hyperpolarizing photocurrents with high spatiotemporal resolution, potentially suggesting it as a powerful platform for neuromodulation, allowing for the exploring of functional connectomes at the sub‐millimeter level.

## 2 Results and Discussion

### 2.1 Optoelectronic Device Fabrication

The present work introduces an optoelectronic biointerface in the form of a photocapacitor, designed to modulate the electrical double layer at the electrode‐electrolyte interface upon irradiation. The photocapacitor consists of thin‐film NUNCD grown on silicon or single crystal diamond substrate, denoted as NUNCD‐Si and NUNCD‐SCD, respectively. NUNCD layers were grown using microwave plasma chemical vapor deposition under nitrogen‐rich atmosphere, as detailed in the Methods section, incorporating n‐type impurities into the NUNCD lattice. The incorporation of nitrogen in the growth of NUNCD serves two pivotal roles. First, the presence of nitrogen in the feed gas facilitates the formation of sp^2^ graphitic grain boundaries within the diamond matrix, significantly enhancing the material's electrical conductivity.^[^ ^22^ ^]^ This enhancement exhibits a strong dependence on the nitrogen concentration in the feed gas, with conductivity increasing markedly, as well described by the Mott‐Davis hopping conduction model.^[^ ^23^ ^]^ Second, nitrogen incorporation modifies the optical absorption properties of UNCD, extending its absorption spectrum to include visible and near‐infrared wavelengths, which are advantageous for certain biological applications. Collectively, nitrogen incorporation transforms UNCD from an insulating material into a semiconducting and electrochemically active platform, establishing NUNCD as a promising candidate for optoelectronic and biointerfacing applications.

**Figure 1:**
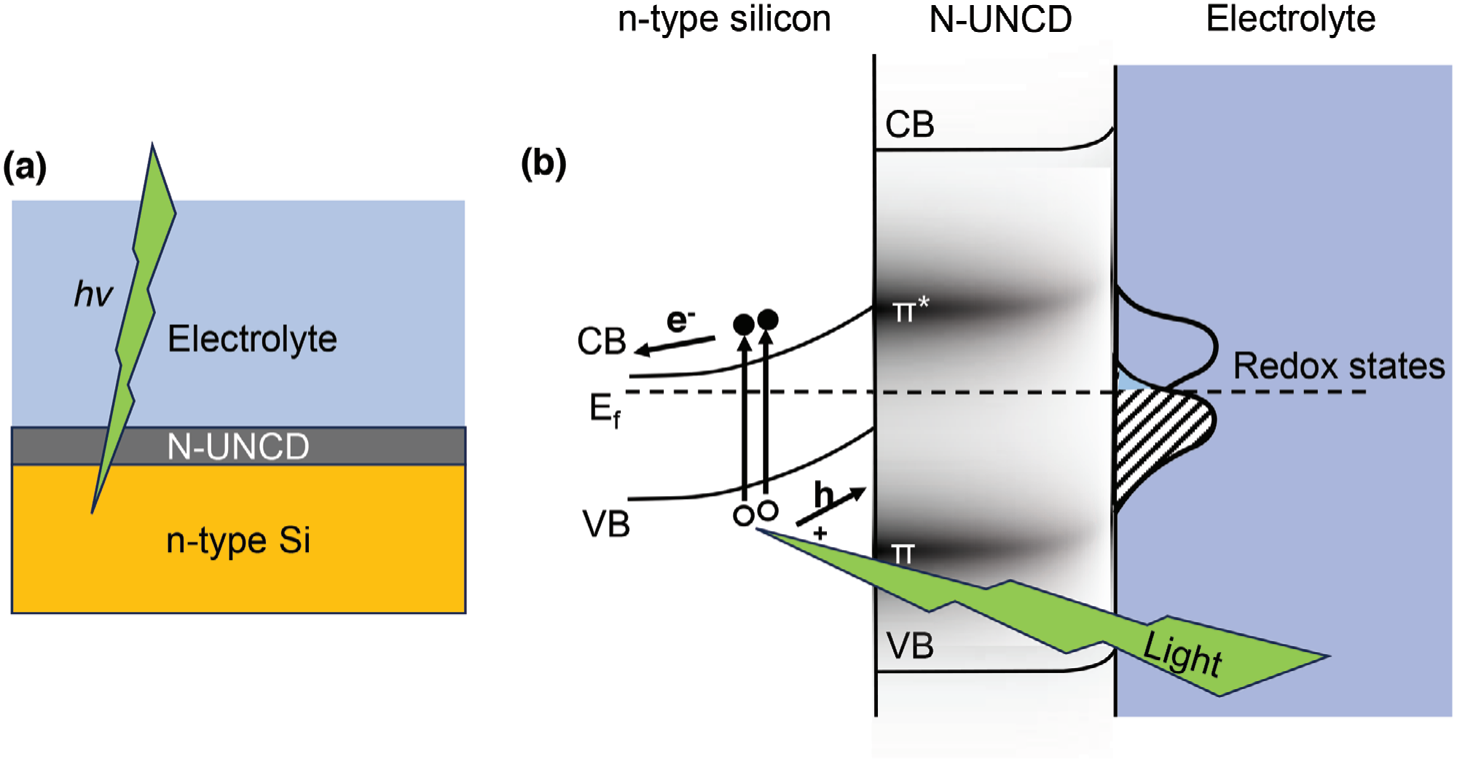
a) Device schematic of NUNCD‐Si. b) Simplified energy band diagram illustrating photoexcitation within n‐type silicon or NUNCD, resulting in a flow of positive photocurrent at the NUNCD‐electrolyte interface. The conductivity of bulk NUNCD was attributed to π and π* sub‐bands within the graphitic grain boundaries, while the surface of the NUNCD was etched by oxygen annealing and rendered non‐conductive. The photocurrent mechanism was capacitive, which was attributed to a redistribution of light‐induced charged carriers at NUNCD‐electrolyte interface.

Experiments were conducted with device immersed in physiological electrolyte (0.15 m NaCl or 1 × phosphate buffered saline) mimicking the osmolarity and ionic strength of cerebral spinal fluid. The NUNCD photoactive layer was exposed to electrolyte via a 2‐mm diameter O‐ring opening. We investigated oxygen surface functionalization, which was known to impart favorable semiconducting properties characterized by a low‐conductivity depletion layer near the surface of the NUNCD.^[^ ^24^ ^]^ X‐ray photoelectron spectroscopy (XPS) of NUNCD in the present work (Figure S1, Supporting Information) showed specific peaks confirming the presence of carbon and oxygen functionalities, which align with previously reported NUNCD spectra, as expected.^[^ ^25^ ^]^ Device schematics (**Figure** **1**a) and the predicted energy band diagram (Figure 1b) illustrate the NUNCD‐Si configuration. Upon optical stimulation, it is thought that photogenerated carriers are produced within the NUNCD and silicon material, subsequently undergoing separation into free carriers. Electrons accumulate at the n‐type NUNCD‐electrolyte interface, resulting in a redistribution of the electrical double layer, which in turn gives rise to transient ionic photocurrent within the electrolyte environment proximal to the device and its illuminated region. The conductive silicon layer serves as a substrate for NUNCD growth, while providing a stable return path in the bioelectronic interface.

### 2.2 Optical Properties

**Figure 2:**
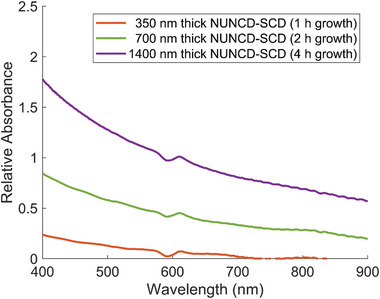
UV–vis absorption spectrum of NUNCD‐SCD biointerface between 400 and 900 nm wavelengths for NUNCD thicknesses of 0, 350, 700, and 1400 nm corresponding to growth times of 1, 2, and 4 h.

The optical properties of NUNCD were analyzed using UV–vis spectroscopy across a wavelength range of 400–900 nm to obtain absorbance spectra relative to NUNCD growth duration. Due to the opaque nature of silicon substrates, absorbance spectra were only measured for NUNCD grown on optically transparent single crystal diamond substrates (NUNCD‐SCD). NUNCD‐SCD exhibited broad absorbance from 400 to 900 nm, offering a wide range of wavelengths for photoexcitation (**Figure** **2**). The strongest absorbance occurred in the UV–vis spectrum (400–450 nm), with moderate absorbance in the visible range (450 – 750 nm) and reduced absorbance in the infrared and beyond (750–900 nm). This trend indicated a stronger absorption of light at shorter wavelength by the NUNCD. Increasing NUNCD growth time, and consequently film thickness, enhanced light absorbance across the entire 400–900 nm spectrum. Herein, we investigated the photovoltage produced using 476 nm light and photocurrent using 595 and 808 nm light. Biological experiments were carried out solely under 595 nm light, as 476 nm wavelength overlapped with the excitation wavelength of Ca^2+^ indicators, used for calcium imaging in this study. The possible contribution to photocurrent by irradiating the silicon beneath the NUNCD merited closer scrutiny. At the wavelength of 595 nm, the measured absorbance of 0.027 for NUNCD‐SCD (1 h growth) corresponded to a transmittance of 93.7%. A similar degree of light transmittance was assumed for NUNCD grown for 1 h on silicon. While light irradiation on the silicon substrate itself produced virtually zero photocurrent (Figure S2, Supporting Information), it is thought that light transmitted through the NUNCD generated free carriers in the silicon, which may subsequently contribute to photocurrent due to charge separation and transport by the NUNCD.

### 2.3 Photoelectrical Properties of Biointerfaces – Two‐Electrode Method

**Figure 3:**
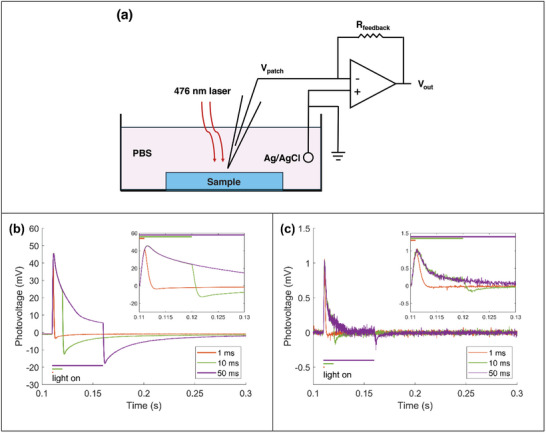
Photoelectrochemical properties of NUNCD‐Si and NUNCD‐SCD biointerfaces. a) Schematic of the two‐electrode “floating” configuration for measuring transient open‐circuit photovoltage. Photovoltage was recorded using an Ag/AgCl electrode within a glass capillary, positioned 2 µm above the device surface, relative to an Ag/AgCl counter electrode. All electrodes were immersed in 1 × PBS, simulating the extracellular environment of neurons in vivo. The device area of 0.038 mm^2^ was exposed to pulsed light (476 nm, 21 mW mm^−2^). b) Photovoltage response for NUNCD‐Si. c) Photovoltage response for NUNCD‐SCD. Photovoltage was measured as the average of 20 traces using an open patch‐clamp pipette (3 MΩ) under light pulses of 1, 10, and 50 ms durations. Horizontal bars indicate the onset and termination of light pulses. Note the difference in the vertical scale between (b) and (c).

Photovoltage at the device‐electrolyte interface was measured using a two‐electrode patch‐clamp setup. This experimental configuration mimicked voltage perturbations encountered by neurons at the surface of the biointerface, thereby simulating the operational characteristics of a standalone implantable device (**Figure** **3**a). The samples used in patch clamp measurements consisted of either NUNCD‐Si or NUNCD‐SCD, with the photoactive NUNCD layer grown for 1 h in each case, resulting in a layer thickness of ≈350 nm. This thickness was optimized to maintain uniform optoelectronic properties across two‐ and three‐electrode measurements. NUNCD‐Si showed asymmetric biphasic photovoltage under pulsed irradiation (476 nm, 21 mW mm^−2^) with pulse widths of 1, 10, and 50 ms (Figure 3b). Upon irradiation, NUNCD‐Si displayed a peak photovoltage of 47 mV and a rise time of ≈2 ms. Similarly, NUNCD‐SCD showed asymmetric biphasic photovoltage with an identical rise time (Figure 3c). However, its peak photovoltage was 1 mV, ≈50 times lower than that of NUNCD‐Si. Such differences between NUNCD‐Si and NUNCD‐SCD may be ascribed to different return electrode resistances and band‐bending effects, which affect the diffusion width and minority carrier diffusion distance. The device consisted of two junctions: NUNCD‐electrolyte and NUNCD‐back contact, where the back contact was either Si or SCD. It was possible that the presence of an electric field at the NUNCD‐Si or NUNCD‐SCD interface caused different degrees of band bending, which in turn impacted the mobility of photogenerated charge carriers.

### 2.4 Photoelectrical Properties of Biointerfaces – Three Electrode Method

**Figure 4:**
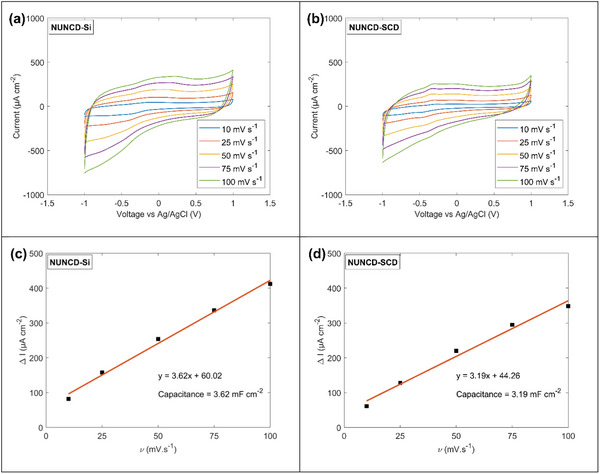
Electrochemical characterization of biointerface in NaCl (0.15 m) with electrode surface area of 3.14 mm^2^. Cyclic voltammetry at various scan rates for: a) NUNCD‐Si. b) NUNCD‐SCD. The capacitance was derived from the slope of linear fit between capacitive current and scan rate in: c) NUNCD‐Si and d) NUNCD‐SCD.

In oxygen‐terminated NUNCD, a double layer at the electrode‐electrolyte interface induces spatial charge separation. Such charge separation establishes a local electric field, which subsequently induces the flow of transient ionic current in the electrochemical medium.^[^ ^26^ ^]^ An equivalent circuit model of NUNCD was reported elsewhere, which fully described the physical mechanisms and the emergence of the electric field at the electrode‐electrolyte interface.^[^ ^24^ ^]^ The electrochemical behavior of the devices was characterized by cyclic voltammetry to quantify capacitive charging. Cyclic voltammograms of NUNCD‐Si (**Figure** **4**a) and NUNCD‐SCD (Figure 4b) exhibited approximately rectangular shapes, which suggested the presence of capacitive behavior, where the current response is predominantly driven by charging and discharging of the electrical double layer, with minimal contribution from Faradaic processes. The enclosed area within the cyclic voltammograms at a scan rate of 100 mV s^−1^ suggested substantial cathodal charge storage capacities for oxygen‐terminated NUNCD‐Si (7.2 mC cm^−2^) and NUNCD‐SCD (9.0 mC cm^−2^) that have undergone 1 h of NUNCD growth and 20 h oxygen surface functionalization. Combined with the wide water window of 2 V, high capacitance of NUNCD‐Si (3.62 mF cm^−2^, Figure 4c) and NUNCD‐SCD (3.19 mF cm^−2^, Figure 4d), these results indicated a high charge injection capacity at the device‐electrolyte interface – important parameters in effective cell stimulation. A sufficient level of charge injection capacity is critical in the context of optoelectronic biointerface for cell stimulation. It enables the delivery of sufficient electrical charge to initiate neuronal firing, broadens the range of stimulation parameters, and minimizes electrode degradation by operating within a smaller voltage window.^[^ ^27^ ^]^

**Figure 5:**
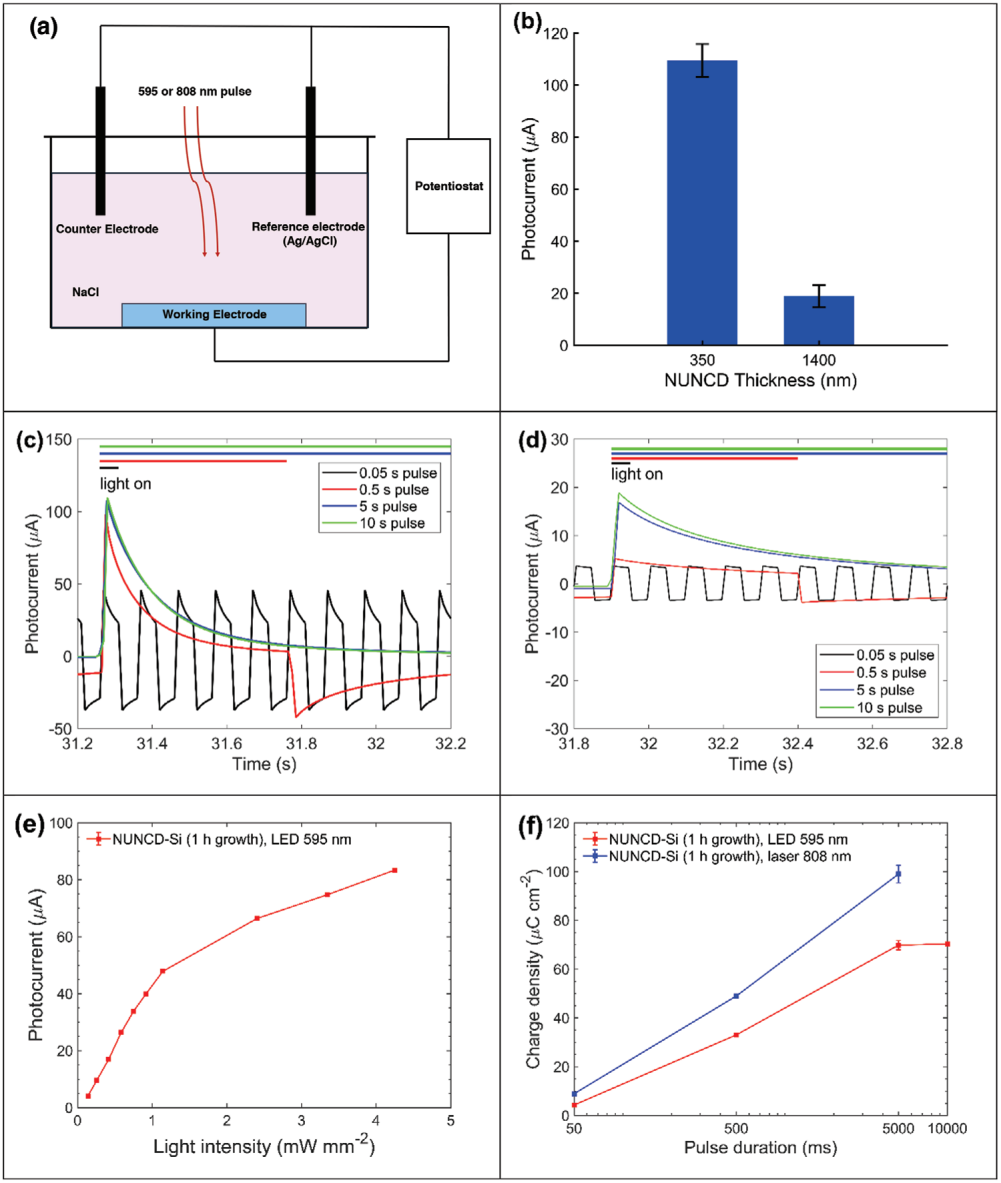
Photoelectrochemical characterization of the NUNCD‐Si biointerface. a) Schematic of the three‐electrode setup, comprising the NUNCD‐Si or NUNCD‐SCD biointerface as the working electrode, a platinum counter electrode, and an Ag/AgCl reference electrode. Short circuit photocurrents were measured between the working and counter electrodes. Unless otherwise specified, light illumination was provided by either an LED (595 nm, 4.3 mW mm^−2^) or a laser (808 nm, 41 mW mm^−2^), with a pulse duration of 0.5 s and a frequency of 1 Hz. The NUNCD‐Si and NUNCD‐SCD were grown for 1 h. b) Photocurrent of NUNCD‐Si as a function of photoactive layer thickness (mean ± s.d, N = 3). Photocurrent characterization for various NUNCD growth durations: c) 1 h d) 4 h. e) Photocurrent under 595 nm LED illumination as a function of light intensity (mean ± s.d., N = 3). f) Charge density of NUNCD‐Si under different light pulse durations (mean ± s.d., N = 3).

The photo‐electrochemical properties of the biointerfaces NUNCD‐Si and NUNCD‐SCD were characterized by measuring photocurrent under 595 and 808 nm wavelengths using three‐electrode configuration (**Figure** **5**a). Two light sources were investigated: yellow LED (595 nm) and near infra‐red laser (808 nm). Photocurrent measurements were conducted in 0.15 m sodium chloride solution to mimic the electrophysiological properties of cerebral spinal fluid (CSF). The optimal thickness of NUNCD was investigated empirically to maximize photocurrent generation in the NUNCD‐Si biointerface. Two thicknesses were investigated using 595 nm irradiation at light intensity of 4.3 mW mm^−2^. At 1 h growth time, NUNCD having a 350 nm thickness exhibited charge‐balanced, capacitive biphasic photocurrents having positive peaks of 109.5, 107.6, 98.1, and 45.7 µA corresponding to optical pulse durations 10, 5, 0.5, and 0.05 s (Figure 5c). The peak photocurrent remained constant as a function of illumination cycles, indicating photochemical stability of NUNCD over 800 illumination cycles (Figure S3, Supporting Information). Furthermore, the photovoltage (Figure S4, Supporting Information) under these conditions matched the profile of the photocurrent. At 4 h growth time, NUNCD having 1400 nm thickness showed peak photocurrents 18.9, 16.9, 5.2, and 3.7 µA (Figure 5d). The capacitive nature of these photocurrents clearly demonstrated a reversible charge injection process. The significance of capacitive charge injection has been widely recognized in mitigating electrode degradation and tissue damage.^[^ ^28^ ^]^ The effect of photoactive layer thickness on the performance of optoelectronic devices, particularly in solar cells, has been extensively studied.^[^ ^29^ ^]^ Generally, a thicker photoactive layer enhances light absorption, resulting in an increased number of light‐generated charge carriers. However, if the layer becomes too thick, it can lead to increased recombination rates within the depletion region, ultimately reducing carrier collection efficiency. Consistent with this trend, our observations suggested that the highest photocurrent was achieved with a photoactive layer thickness of 350 nm, corresponding to 1 h of growth. In comparison, as the thickness increased to 1400 nm, or 4 h of growth, the photocurrent decreased (Figure 5b).

In the context of neuronal stimulation, the depolarization and repolarization of neurons depend on charge density, specifically the integrated photocurrent over time, which must exceed a critical threshold for different neural cell types.^[^ ^30^ ^]^ In this study, the observed biphasic capacitive photocurrent supported both depolarization and hyperpolarization of neuronal membrane potentials. The photocurrent from NUNCD‐Si increased with light intensity across all tested levels (Figure 5e). In addition, we quantified the total charge generated at biointerfaces under 595 and 808 nm light by integrating the area under the photocurrent curve. For excitatory photocurrent generation, NUNCD‐Si illuminated with 595 nm LED produced charge densities of 4.3, 33.1, 69.9, and 70.3 µC cm^−2^ for optical pulse durations of 50, 500, 5000, and 10 000 ms, respectively (Figure 5f). The wide range of charge densities achieved by varying pulse widths meet the threshold requirements for typical human neural prostheses, including epi‐retinal stimulation (5–570 µC cm^−2^),^[^ ^31^ ^]^ optic nerve stimulation (4–62 µC cm^−2^),^[^ ^32^ ^]^ and cortical stimulation (11 µC cm^−2^).^[^ ^33^ ^]^

**Figure 6:**
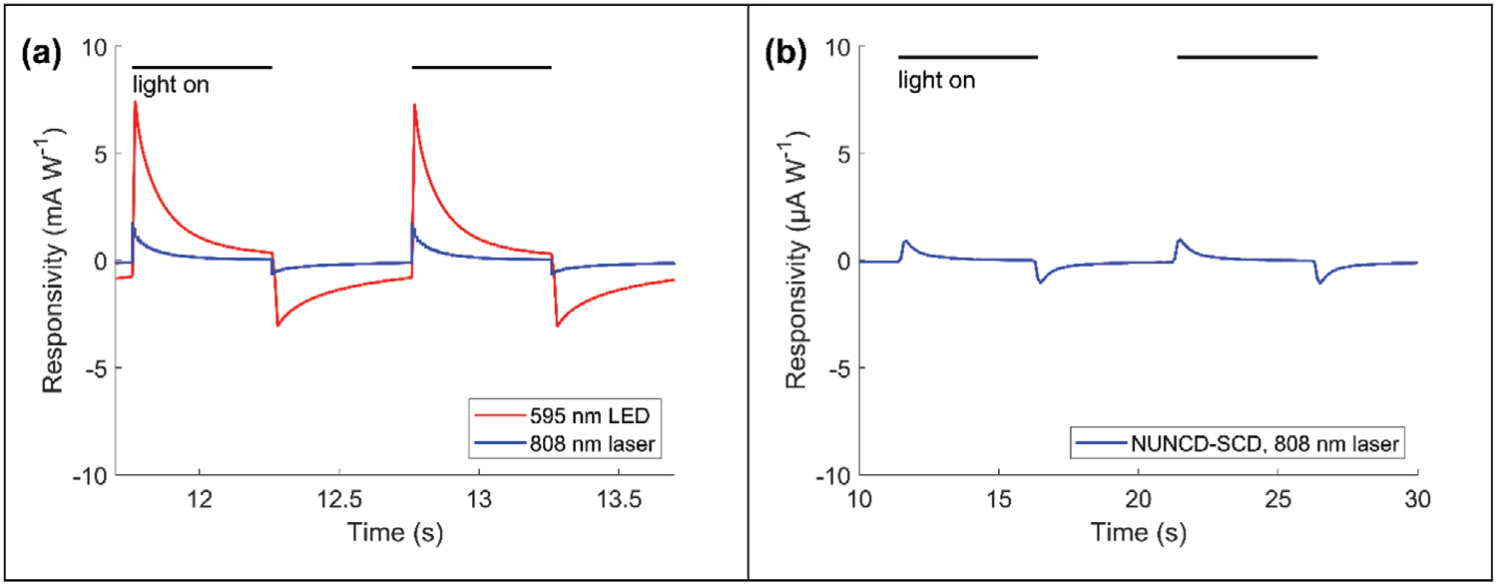
Comparison of photocurrents between NUNCD‐Si and NUNCD‐SCD. a) Photocurrents of NUNCD‐Si under 0.5 s light pulses at 1 Hz, with illumination at 595 nm (4.3 mW mm^−2^) and 808 nm (41 mW mm^−2^). b) Photocurrents of NUNCD‐SCD under 5 s light pulses at 0.1 Hz, with illumination at 808 nm (41 mW mm^−2^). Photocurrent could not be reliably measured in NUNCD‐SCD under 595 nm LED illumination, due to low photoresponse to light intensity of 4.3 mW mm^−2^. Black bars indicate when light was on.

NUNCD‐Si demonstrated a large photoresponse, exhibiting biphasic current waveforms with photocurrent density of ≈1.5 mA W^−1^ under 808 nm light and 7.5 mA W^−1^ under 595 nm light (**Figure** **6**a), suggesting enhanced quantum efficiency of the NUNCD‐Si biointerface at a lower wavelength. When switching the light with a pulse width of 0.5 s at a frequency of 1 Hz, the photocurrent peaked within 10 ms. This rapid charging kinetics of NUNCD‐Si suggests promising potential for application in neuromodulation, as the firing rates of cortical neurons typically range from 1 to 20 Hz.^[^ ^34^ ^]^ **Table** **1** provides a summary of recent semiconducting optoelectronic devices used for neural photostimulation. The photoresponsivity of NUNCD‐Si reported in this study is 7.5 mA W^−1^, which is comparable to devices reported elsewhere, such as InP/ZnO quantum dots (2.2 mA W^−1^),^[^ ^11^ ^]^ Cr/AU:H_2_Pc:PTCDI (6.6 mA W^−1^),^[^ ^9b^ ^]^ and CdSe/CdS nanorods (0.6 mA W^−1^),^[^ ^10^ ^]^ albeit lower than P3HT‐PCBM with a PEDOT:PSS coating (30 mA W^−1^).^[^ ^35^ ^]^ These devices successfully induced excitatory neural modulation in SH‐SY5Y cells, primary hippocampal neurons, and in light‐insensitive rat and chick retinas, respectively. In contrast, the photocurrent density in NUNCD‐SCD was ≈0.89 µA W^−1^ under 808 nm light (Figure 6b), which was lower than that in NUNCD‐Si by approximately three orders of magnitude.

**Table 1:** Comparison of optoelectronic biointerfaces for neural photostimulation. The responsivity of NUNCD‐Si in this work is similar to semiconducting devices reported by others that are capable of excitatory neuromodulation.

Optoelectronic device material	Cell type	Wavelength [nm]	Irradiation intensity [mW mm^−2^]	Dominant charge generation mechanism	Responsivity [mA W^−1^]	References
NUNCD‐Si	Light‐insensitive rat retinas	595	4.3	Capacitive	7.5	Present work
P3HT‐PCBM	Rat hippocampal neurons	532	10	Capacitive	N/A	Ghezzi et al.(2011)^[^ ^36^ ^]^
P3HT‐PCBM with PEDOT:PSS	SH‐SY5Y	445	1	Capacitive	30	Han et al.(2020)^[^ ^35^ ^]^
InP/ZnO QD	Primary hippocampal neurons	445	0.57	Faradaic	2.2	Karatum et al.(2021)^[^ ^11^ ^]^
Cr/Au/H_2_Pc/ PTCDI	Light‐insensitive rat retinas	660	10.8–17.3	Capacitive	6.6	Rand et al.(2018)^[^ ^9b^ ^]^
CdSe/CdS	Light‐insensitive embryonic chick retinas	405	0.7	Capacitive	0.6	Bareket et al.(2014)^[^ ^10^ ^]^

### 2.5 Calcium Imaging

**Figure 7:**
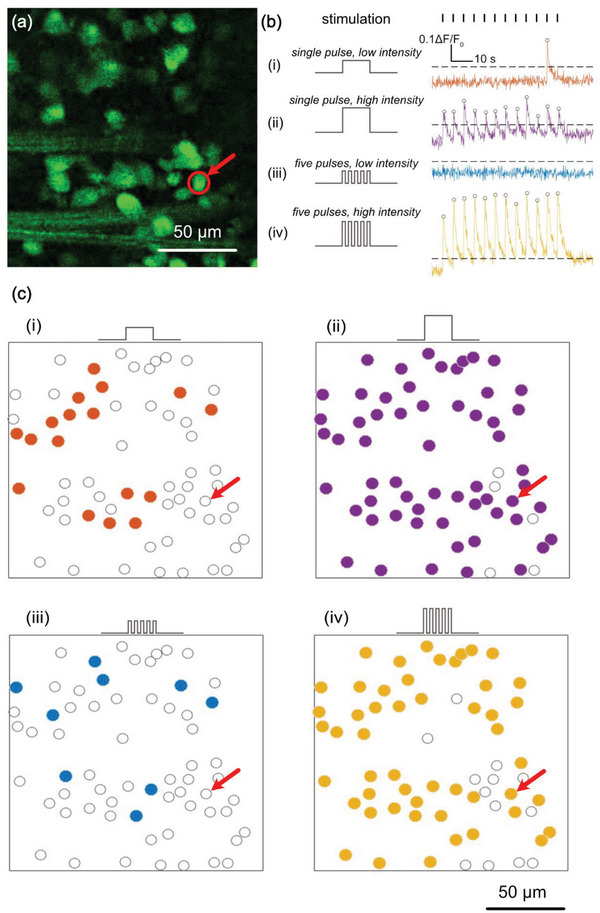
Optoelectronic stimulation of OGB‐1‐labeled RGCs on an oxygen‐terminated NUNCD‐Si biointerface. a) Schematic of time‐lapse calcium imaging, where somas were identified. Calcium transients from a representative cell (indicated by a red circle and an arrow) are shown in (b). Four different stimulation protocols were tested using 595 nm light: conditions (i) and (ii) involved single pulses of 500 ms duration, while conditions (iii) and (iv) used five consecutive pulses of 50 ms duration each. Conditions (i) and (iii) were delivered at 1.1 mW mm^−^^2^, while (ii) and (iv) at 4.3 mW mm^−^^2^. Each trial was repeated 12 times with a period of 5 s. The stimulation onset was shown using black bars. Cellular responses were considered significant if calcium transients exceeded a threshold (black dashed line, see methods) within 1.5 s of stimulus onset, with significant responses labeled by circles in the calcium transient traces. Cells were classified as “activated” if they responded in over 50% of trials. Activation patterns for each condition were shown in (c), with activated cells represented by colored filled circles and inactive cells by open circles. The example cell was marked using red arrows in (c).

Calcium imaging experiments were conducted using Royal College Surgeon (RCS) rat model of retinal dystrophy in explanted retinas at 90 days postnatal. As expected, RCS rats exhibited no intrinsic response to light due to inherited retinal degeneration. To reduce light‐induced artifacts in the calcium dye, pulsed light was delivered to the biointerface through an optical fiber positioned ≈350 µm from the imaging field of view (Figure S5, Supporting Information). We recorded activities from 64 RGCs and their responses to photoelectrochemical stimulation within a 159 × 159 µm^2^ region (**Figure** **7**a). Photoexcitation of NUNCD‐Si using pulsed light stimulation (595 nm, 12 pulses, 500 ms pulse duration, 5 s intervals) resulted in upward deflections in fluorescence (ΔF/F_0_) within RGCs at a representative time stamp (t_1_ = 10.3 s), with subsequent responses occurring at 5‐second intervals. Two intensities of 1.1 and 4.3 mW mm^−2^ (Figure 7b,c,‐i, ii) were used, which led to different cellular responses. The expected photocurrents under these conditions were 47.9 and 83.4 µA, as previously shown (Figure 5e).

Furthermore, using shorter pulse trains at the same wavelength (595 nm), consisting of 12 trains with five consecutive pulses each (50 ms pulse duration and 5 s intervals), produced discernible calcium oscillation at both 1.1 and 4.3 mW mm^−2^ and different activation patterns (Figure 7b,c‐iii, iv). In both illumination schemes, when cells were activated, the rapid increase in ΔF/F_0_ was followed by a gradual return to baseline levels. Collectively, these results demonstrate effective optoelectronic stimulation through the NUNCD‐Si biointerface, as evidenced by the increase in calcium concentration upon illumination and the subsequent return to resting intracellular calcium levels, indicating neuronal activity. At 4.3 mW mm^−2^ (Figure 7c‐ii, iv; Videos S2 and S4, Supporting Information), a greater number of neurons within the same imaging FOV exhibited activity compared to 1.1 mW mm^−2^ (Figure 7c‐i, iii; Videos S1 and S3, Supporting Information), suggesting that higher photocurrents and charge densities activated more neurons.

Using an uncoated silicon wafer that did not have any NUNCD growth, we demonstrated an absence of calcium flux in the retinas, as there was insufficient photocurrent produced to induce neuronal firing (Figure S6, Supporting Information). If light‐induced thermal effect were to induce neuronal firing via triggering temperature‐sensitive ion channel, calcium flux would be observed in this inactive device. Thus, the absence of calcium flux in the inactive device confirmed that neuronal activation was attributed to photocurrent from NUNCD rather than any photothermal effects.

### 2.6 Optical Safety Threshold

The American National Standards Institute (ANSI) Z136.1‐2000 Standard for ocular safety defined the maximum permissible radiant power (MPΦ) under pulsed light exposure as: *MP*∅  =  6.93 × 10^−4^ *C_T_C_E_t* ^−0.25^, where C_T_ and C_E_ are wavelength‐dependent parameters and t denoted time in seconds.^[^ ^37^ ^]^ For 595 nm light, C_T_ = 1. C_E_ was governed by angular spread of the incident beam and was 29.3 W mm^−2^ for retinal diameter >1.7 mm. In the present study, light pulse durations of 50 and 500 ms yielded corresponding MPΦ of 9.8 and 5.5 mW, respectively. Consequently, the light intensities investigated using the 595 nm LED (1.1 and 4.3 mW mm^−2^) having pulse durations between 50 and 500 ms, were within the safe threshold for ocular stimulation.

### 2.7 Significance and Limitations

We demonstrate the use of a diamond‐based photoelectrode biointerface to stimulate RGCs in degenerate rat retinas through photovoltaic methods. Our findings show that the magnitude of ionic photocurrent used for stimulation can be adjusted by varying the thickness of the NUNCD photoactive layer, as well as by changing pulse duration and irradiance. These parameters allow for versatile stimulation options while maintaining physiologically safe light intensities. The NUNCD‐Si biointerface is compact (5 × 5 × 0.3 mm^3^), untethered, and features a straightforward device architecture. These properties suggest that it holds promise for simplifying surgical procedures in implantable photoelectric stimulation prosthetics.

However, our study has two notable limitations. First, the biointerface's high stiffness and low compliance hinder its ability to conform to neural tissue, which could lead to inflammation, scarring, and functional loss due to the mismatch in stiffness between the diamond surface (in the GPa range) and soft brain tissue (in the kPa range).^[^ ^38^ ^]^ To address these issues, one approach could be to develop flexible substrates for the NUNCD film. For example, curved silicon membranes have been designed to conform to spherical shapes by creating mechanically and electrically interconnected islands using techniques such as photolithography and reactive ion etching.^[^ ^39^ ^]^ Another strategy involves applying organic coatings such as PEDOT:PSS, which have been shown to enhance charge transfer and reduce impedance in neural electrodes.^[^ ^40^ ^]^

The second limitation concerns the spatial resolution and selectivity of stimulation. In the present study, the NUNCD‐Si biointerface stimulates only the regions directly exposed to light, with spatial selectivity constrained by the resolution of the LED, which in the present setup is in the order of hundreds of micrometers. Given that neurons are ≈20 µm in diameter, the present resolution does not permit single‐cell or subcellular stimulation. Future research could focus on enhancing spatial resolution and selectivity by fabricating micro‐ or nanoscale pillar arrays on the silicon substrate using photolithography and reactive ion etching.^[^ ^41^ ^]^ This approach would allow for high spatial control by enabling each pillar to serve as a localized site for electrical charge, thus improving the precision of photovoltaic stimulation.

## 3 Conclusion

In the present work, the NUNCD‐Si device operated wirelessly and under a broadband visible spectral region spanning from blue to the infra‐red wavelengths. Electrochemistry in NUNCD‐Si revealed high capacitive photocurrent (100 µA) and charge density (70.3 ± 0.8 µC cm^−2^) using 595 nm wavelength at low light intensities suitable for neural stimulation (1.1–4.3 mW mm^−2^). The charging and discharging of capacitive photocurrent in the NUNCD‐Si, within a highly precise and confined region of illumination, can be controlled by switching the light source on or off. The photocurrent response can be modulated by the wavelength and pulse duration of optical stimulation as well as by the thickness of the photoactive NUNCD, providing a versatile set of parameters to modulate transmembrane depolarization with high spatiotemporal resolution. The biphasic nature of capacitive photocurrent allowed for control of depolarization and hyperpolarization events across neuron's cell membrane, offering a route for optoelectronically modulated neurostimulation. The photoresponse in NUNCD‐Si was comparable to the performance of optoelectronic devices reviewed earlier, where successful extracellular neurostimulation was accomplished. Functional studies using Ca^2+^ demonstrated successful neurostimulation of blind retinas immersed in physiological medium. Here, irradiation of NUNCD‐Si using 595 nm wavelength consistently produced synchronized calcium oscillations in neurons, demonstrating the efficacy of NUNCD‐Si biointerface to elicit neuronal activities optoelectronically. The wireless nature and inherent biocompatibility of NUNCD‐Si biointerface pave a promising platform for future development of non‐genetic, minimally invasive optoelectronic neurostimulator for enhanced optical control of neural activity.

## 4 Experimental Section

### Biointerface Fabrication

The biointerface was fabricated by growing NUNCD on either Si or SCD substrate. Si (n‐type, 5 × 5 × 0.5 mm^3^) and SCD (4 × 4 × 0.3 mm^3^, RH443PPFF, EDP Corporation) were cleaned in acetone, isopropanol, and double distilled water under gentle sonication to remove surface contaminants. The method for growing NUNCD has been reported elsewhere.^[^ ^19^ ^]^ Briefly, NUNCD growth was carried out inside a microwave plasma chemical vapor deposition system (iPLAS) with a gas mixture of 79% argon, 20% nitrogen, and 1% methane (BOC Australia, purity 99.999%) at pressure of 80 Torr. Si and SCD substrates were placed on top of 850 °C heating stage and grown for 1 or 4 h. Samples were subsequently surface‐terminated by annealing in oxygen inside a vacuum furnace (OTF‐1200X, MTI Corp) at 420 °C for 20 h.

### Ultraviolet‐Visible Spectroscopy

UV–vis spectroscopy (SPECORD 250 PLUS, Analytik Jena) was performed on solid‐state N‐UNCD/SCD using a custom 3D printed holder. The holder was affixed to the spectrometer and allowed light passing through a 2 × 2 × 0.3 mm^3^ sample mounted in the center of the holder. The absorbance by the sample was measured for wavelengths ranging from 400 to 900 nm.

### Surface Chemistry Characterization

XPS was performed to determine the surface elemental composition of NUNCD films. Data were acquired using a K‐Alpha system (ThermoFisher) with a monochromated Al Kα source (1486.7 eV) with pass energy of 20 eV.

### Photoelectric Characterization

The open‐circuit photovotage was recorded in two‐electrode setup, via a glass patch pipette filled with 1× phosphate buffered saline (PBS) connected to a patch clamp amplifier (SEC‐05X, NPI). Pulsed blue light irradiation (476 nm, part number, Thorlabs) was applied at 0.5 Hz frequency and 1, 10, and 50 ms pulse width. The glass patch pipette was positioned near the sample's surface (<2 µm). Biointerfaces NUNCD‐Si and NUNCD‐SCD were placed inside the recording chamber and fully immersed in PBS. A 476 nm optogenetic laser (OptoTech) acted as the source of optical stimulation. An optical fiber (M132L02, Thor Labs) guided the laser through the top port of an upright confocal microscope (FV1200, Olympus), through a 40× objective and an additional 4× magnification camera lens. A laser spot size of 220 µm or 0.038 mm^2^ was observed in the image plane. Light exiting the objective was measured using a power meter (FieldMaxII‐TO, Coherent).

A potentiostat (Gamry Interface 1000E) was used to measure the photocurrent in the biointerface, i.e., NUNCD‐Si or NUNCD‐SCD, in three‐electrode configuration. The backside of the biointerface was connected directly to the working electrode (WE). The platinum counter electrode (CE) and Ag/AgCl reference electrode (RE) were used. Photocurrent measurements were conducted inside ionic medium NaCl (0.15 m). An area of biointerface (3.14 mm^2^) was exposed to the electrolyte via the interior of a 2‐mm diameter O‐ring. The O‐ring was secured in place by applying downward pressure on the recording chamber via four mechanical screws, providing a tight seal against any potential leakage of electrolyte from the chamber. LED (595 nm, M595F2, Thorlabs) and laser diode (808 nm, Wuhan Lilly Electronics) were used as the illumination sources. The pulse width and light intensities were controlled using a custom Matlab program. Optical powers were measured using a power meter (FieldMaxII‐TO, Coherent).

### Cyclic Voltammetry

Cyclic voltammetry measurements were performed using a potentiostat (Gamry Interface E1000) and the accompanying software. The NUNCD‐Si device was immersed in NaCl (0.15 m), where current was measured by subjugating the device to applied potential window between −1 to +1 V using a scan rate of 100 mV.s^−1^ for six cycles, relative to a silver/silver chloride (Ag/AgCl) reference electrode. The potential limits were selected to prevent irreversible reactions beyond the water window.

### Retinal Preparation

All experimental procedures were approved by the University of Melbourne Animal Ethics Committee (Ethics Approval #27 274) and conformed to the policies of the National Health and Medical Research Council of Australia (NHMRC).

RCS rats at 3 months of age were anesthetized, enucleated, and sacrificed using an overdose of Letharbarb via intracardiac injection. A small incision was made at the optic nerve ending, where calcium indicator OGB‐1 (1 µL, 20 mm, Invitrogen) was injected, as previously described.^[^ ^42^ ^]^ Following dye injection, the lens and cornea were carefully removed. The retinas were maintained at room temperature and in the dark in carbogenated Ames’ solution overnight. On the next day, each retina was mounted on the samples (NUNCD‐Si and NUNCD‐SCD) with the RGCs facing upward for imaging. A stainless‐steel harp was positioned over the retina to minimize movement. During experiments, retinas were superfused continuously in Ames’ solution at rate of 5–8 mL min^−1^ at room temperature.

### Calcium Imaging

Calcium imaging was performed on retinas derived from RCS rats as described above. Confocal microscope (FluoView FV1200, Olympus) outfitted with 40× objective lens was used to acquire fluorescent images over time within a 159 × 159 µm field of view. During recording, RGCs were excited electrically by irradiating NUNCD‐Si biointerface under pulsed 595 nm light. Four illumination protocols were used: i) 500 ms single pulse, 1.1 mW mm^−2^, 5 s intervals; ii) 500 ms single pulse, 4.3 mW mm^−2^, 5 s intervals; iii) five consecutive pulses of 50 ms duration each, 1.1 mW mm^−2^, 5 s intervals; iv) five consecutive pulses of 50 ms duration each, 4.3 mW mm^−2^, 5 s intervals. Light was delivered using a 400 µm diameter optical fiber (FT400EMT, 0.39 NA, Thorlabs) positioned on the retinal surface. The lateral distance (i.e., in the XY plane) between the tip of the optical fiber and imaging field of view was 350 µm. Time series of calcium imaging was performed under excitation and emission of 488 and 525 nm, respectively. Data was captured from a single retina at a frequency of 7.8 Hz.

### Data Analysis

Time lapse images were analyzed using ImageJ (NIH) and Matlab (Mathworks). For each recorded field of view, regions of interests (ROIs) were identified based on the standard deviation of the recorded images over time, and manually selected. The normalized change in fluorescence, ΔF/F_0_ = (F‐F_0_)/F_0_, was calculated where F represented the instantaneous fluorescence and F_0_ was the average fluorescence during the 1–2 s preceding light irradiation. Cellular responses were considered significant if calcium transients exceeded a threshold within 1.5 s of stimulus onset. The threshold was determined as four times the standard deviation of the normalized calcium transient during the 1–2 s preceding light stimulation. Cells were classified as “activated” if they responded in >50% of trials.

### Ethical Statement

All animal experiments were approved by the University of Melbourne Animal Ethics and Welfare Committee (ethics ID 27 274). These procedures complied with the Australian National Health and Medical Research Council code of practice for the use of animals in research.

## Conflict of Interest

S.P. was a former shareholder in iBIONICS, a company focusing on vision restoration using diamond‐based retinal implant. S.P. is a shareholder and public officer of Carbon Cybernetics Pty Ltd., an Australian company specializing in the development of advanced brain‐machine‐interface using carbon‐based devices. A.A. is a shareholder of BrainConnect Pty Ltd., an Australian startup company developing novel solutions to interface the brain and body to treat neurological disorders.

## Author Contributions

Y.Y. performed conceptualization and design of experiments, device fabrication and characterization, calcium imaging, and its data analysis, writing of the original manuscript, review, and editing of the manuscript, and visualization. A.A. performed conceptualization and design of experiments, review and editing of manuscript. A.C. performed drawing energy band diagram, review, and editing of the manuscript. W.T. performed conceptualization and design of experiments, preparation of retinas, calcium imaging and its data analysis, review, and editing of the manuscript. S.P. performed conceptualization and design of experiments, funding acquisition, supervision, review, and editing of the manuscript. The manuscript was written through contributions of all authors. All authors have given approval to the final version of the manuscript.

## Supporting information



Supporting Information

Supplemental Video 1

Supplemental Video 2

Supplemental Video 3

Supplemental Video 4

## Data Availability

The data that support the findings of this study are available from the corresponding author upon reasonable request.
