# Supplementary material for: Nitrogen‐Doped Ultrananocrystalline Diamond – Optoelectronic Biointerface for Wireless Neuronal Stimulation
Source: Adv Healthc Mater. 2025 Feb 11;14(9):2403901. doi: 10.1002/adhm.202403901 (PMC11973940; doi:10.1002/adhm.202403901)
Supplement: Supplementary file 1 — Supporting Information [file ADHM-14-0-s004.docx]

Supporting Information

**Nitrogen-doped Ultrananocrystalline Diamond – Optoelectronic Biointerface for Wireless Neuronal Stimulation**

Yue Yao^*^, Arman Ahnood, Andre Chambers, Wei Tong, Steven Prawer^*^


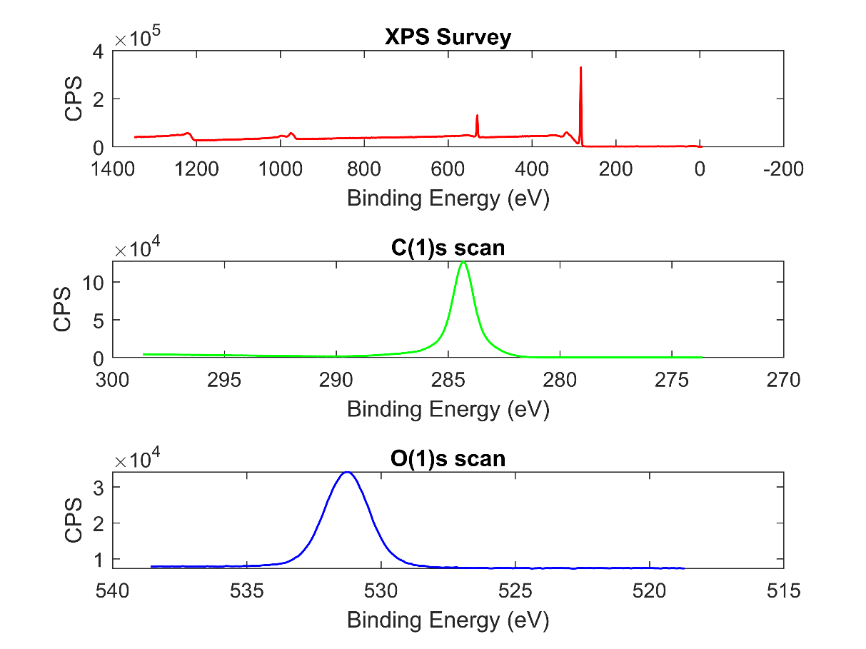


**Figure S1.** XPS spectra for NUNCD-Si. NUNCD-Si was grown for 1 h and subsequently oxygen annealed for 20 h. (A) Survey. (B) C 1s. (C) O 1s.

| **(a)**  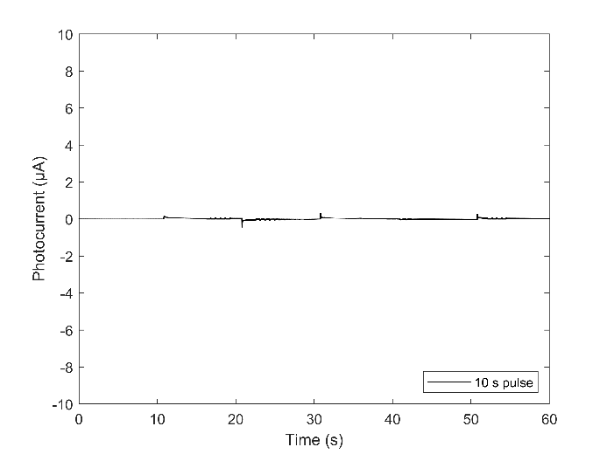 | **(b)**  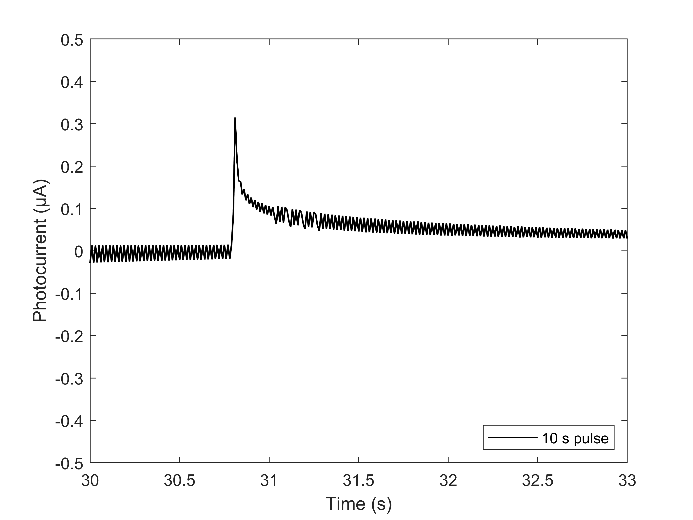 |
| --- | --- |

**Figure S2.** Photocurrent response in 0.15 M NaCl for (a) uncoated n-type silicon and (b) magnified view of the photocurrent peak. Light source, LED (595 nm, 4.3 mW mm^-2^). These results suggest that in the absence the NUNCD layer, the contribution to photocurrent by the uncoated n-type silicon substrate itself was negligible.

***
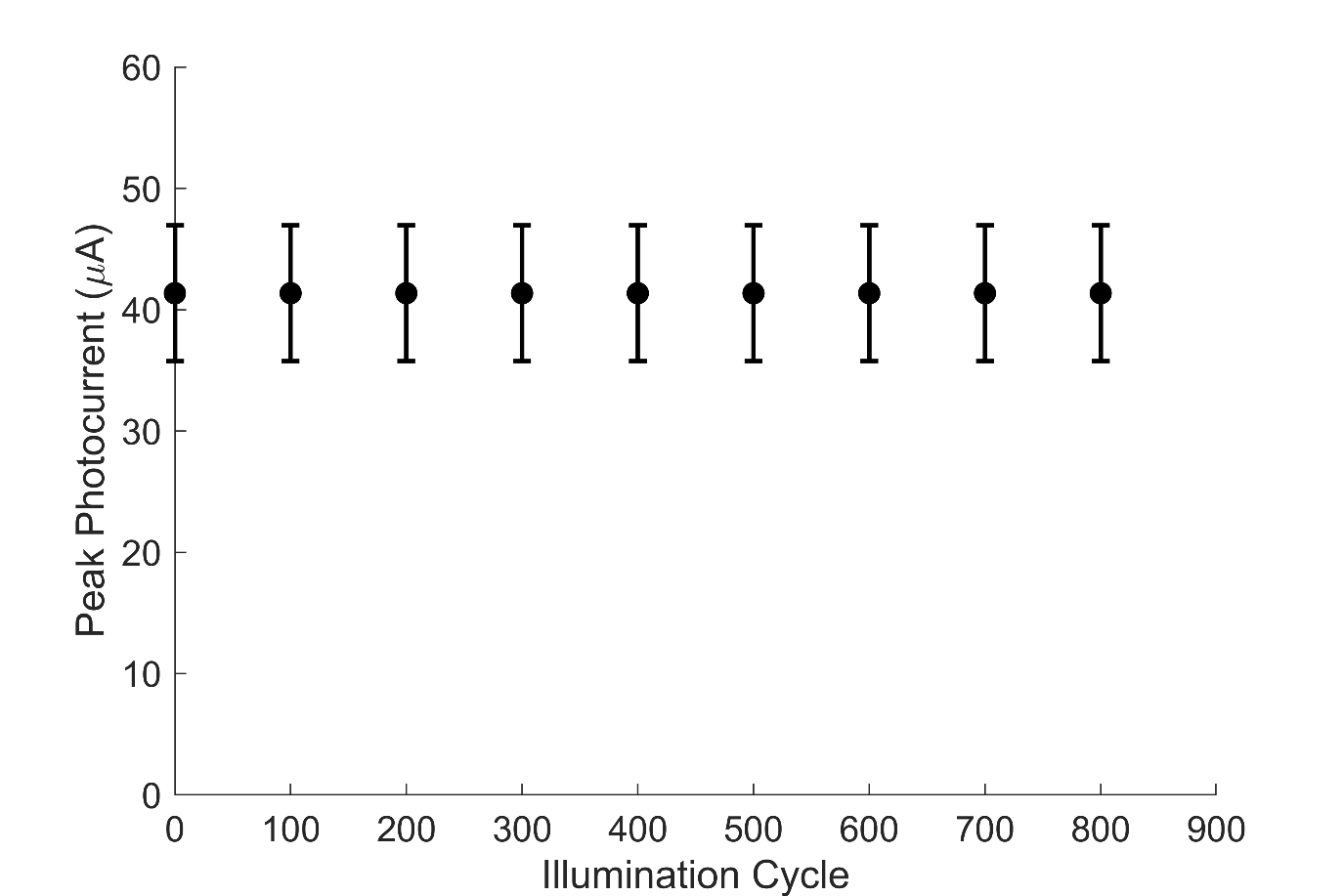
***

**Figure S3.** Cyclic photostability test for NUNCD in 0.15 M NaCl under pulsed illumination (595 nm, 4.3 mW mm^-2^). The peak values of photocurrent over 800 illumination cycles (mean ± s.d, N = 3).


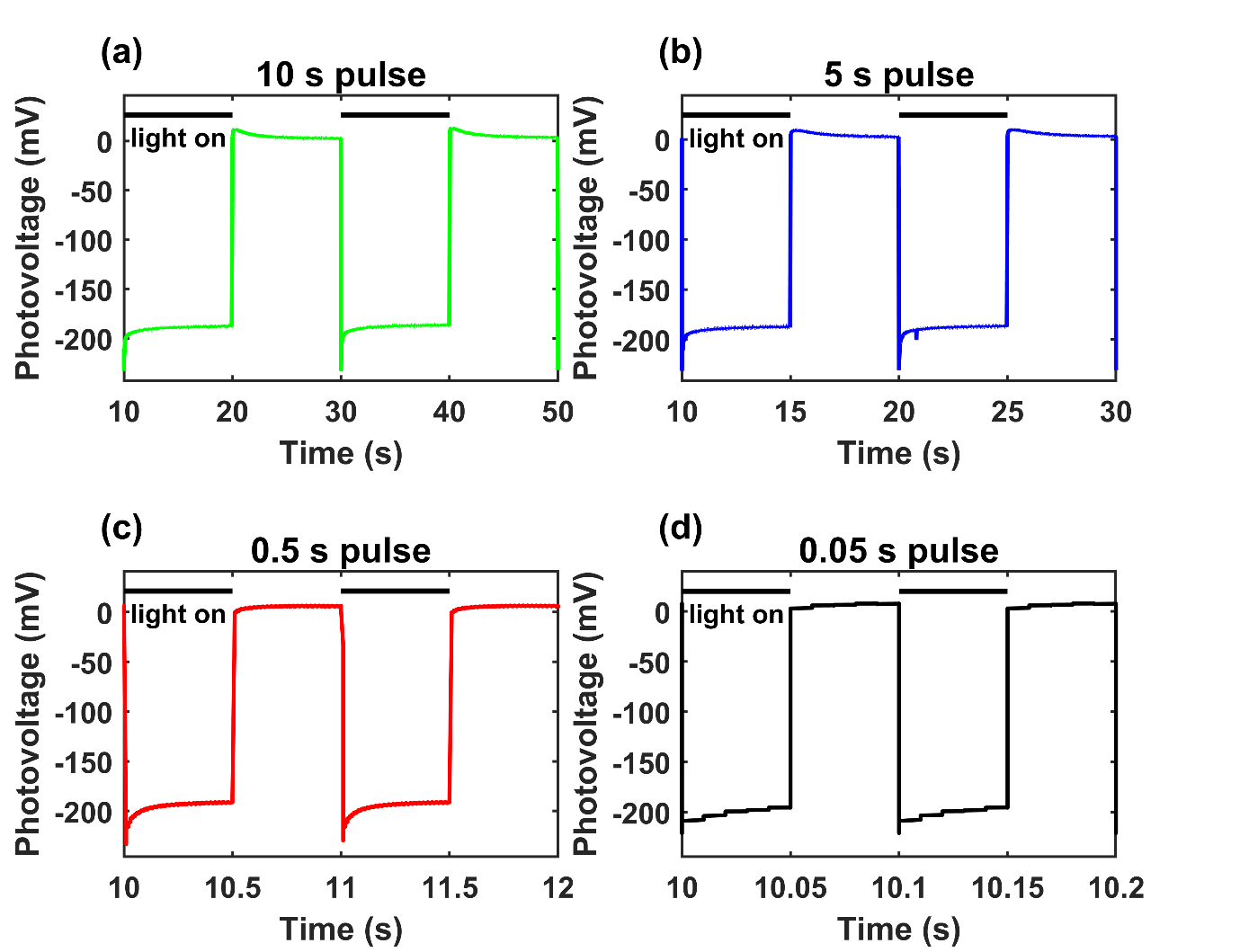


**Figure S4.** Characterization of photovoltage in NUNCD-Si using three-electrode configuration. Photovoltage was measured under a 595 nm pulsed light illumination with 4.3 mW mm^-2^ intensity and various pulse durations. (a) 10 s pulse. (b) 5 s pulse. (c) 0.5 s pulse. (d) 0.05 s pulse.

**Figure S5.** Maximum intensity projection image of the retina showing the location of optical fibre (dashed circle) relative to Ca^2+^ imaging field of view (dashed square). The optical fibre was positioned 350 µm away from the center of the imaging field of view. Irradiation from LED (595 nm, 1.1 or 4.3 mW mm^-2^) was delivered via optical fibre onto horizontally oriented axon bundles in connection with neurons. Scale bar, 50 µm.

| **** |
| --- |
| **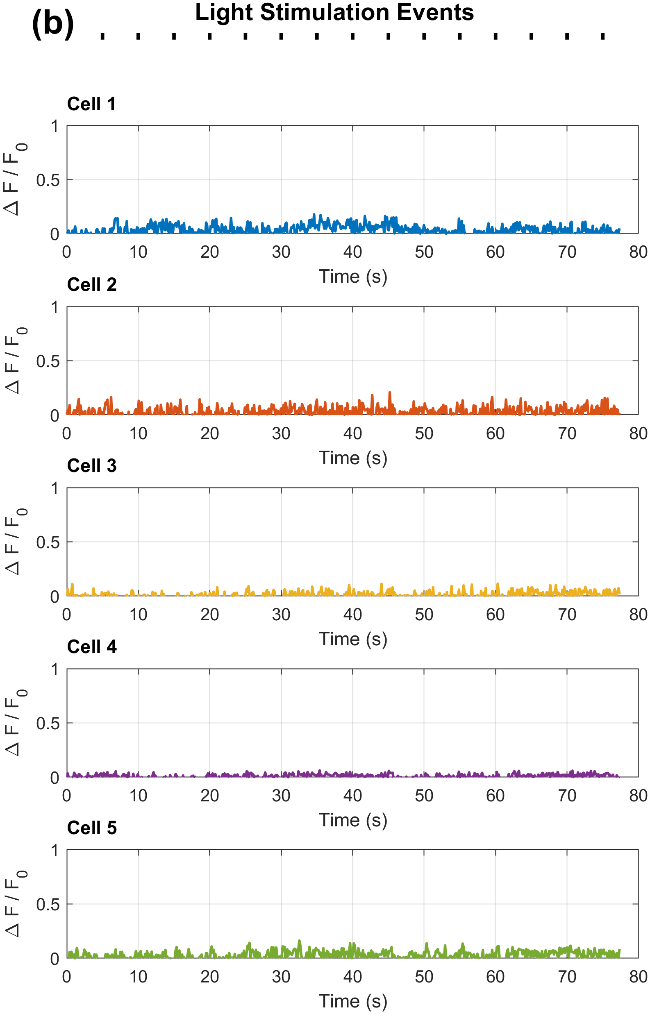** |

**Figure S6.** Calcium transients from representative cells in inactive sample comprised of n-type silicon without NUNCD coating. (a) Schematic of time-lapse calcium image showing somas of presentative cells. (b) Calcium transients. Light irradiation consisted of single pulses of 500 ms duration using 595 nm light at intensity 1.1 mW mm^-2^. Light stimulation events were marked using black bars.
